# Bipolar Disorder after COVID-19 Infection: A Case Report from an Ethiopian Perspective

**DOI:** 10.1155/2022/8931599

**Published:** 2022-08-31

**Authors:** Elias Tesfaye, Selamawit Alemayehu, Elias Gebru

**Affiliations:** ^1^Jimma University Medical Center, Ethiopia; ^2^St. Paul Hospital Millennium Medical College, Ethiopia

## Abstract

**Introduction:**

COVID-19 has been a sudden public health crisis since January 2020, spreading from the city of Wuhan, China, to the whole country within a month and posing serious threats to lives. The pandemic has a profound effect on all aspects of society, including mental health and physical health. The actual effect of the virus on the brain and possible psychiatric manifestations is still an area of study and further investigation. There are also several case reports showing manic like symptoms after COVID-19 infection. We describe the case of a 55-year-old patient who presented with behavioral and mood symptoms after a COVID-19 infection. *Case Presentation*. The patient presented with behavioral disturbance after a diagnosis of COVID-19. He exhibited symptoms including irritability, verbal and physical aggressiveness, increased goal-directed activity, elated and expansive mood, increased energy, grandiosity and inflated self-esteem, and decreased need for sleep. Findings on psychiatric evaluation encompassing detailed history and mental state examination suggested bipolar disorder due to COVID-19 infections. For this, he was put on sodium valproate 1000 mg per day and later, and he was discharged after 21 days with improvement.

**Conclusions:**

This case highlights the importance of paying attention to psychiatric symptoms in patients with COVID-19 and the early intervention and involvement of psychiatrists especially in critically ill patients. In the present scenario, we urge physicians to pay attention to those cases and be open-minded for such a possible new diagnosis. We also recommend performing antibody tests for CSF and RNA tests for patients with mental abnormalities following COVID-19. Further studies can be performed to identify the relationship between COVID-19 and bipolar disorders.

## 1. Introduction

COVID-19 is a deadly pandemic that has overwhelmed numerous healthcare systems across the globe. The outbreak of the new coronavirus (also called COVID-19) since January 2020 is a sudden public health crisis that spread from the city of Wuhan, China, to the whole country within a month, posing serious threats to lives [1, 2].

The fact that COVID-19 is more transmissible and has a case-fatality rate (2.3%) is substantially higher than that for seasonal influenza [3]. In addition, the case fatality is higher reaching up to 8% in elderly and immunocompromised patients. Most existing evidence is uncertain about the incubation period of the virus, and its possible asymptomatic transmission causes additional fear and anxiety [3]. In Ethiopia, there have been 493,167 infections and 7,571coronavirus-related deaths reported in the country since the pandemic began [4].

The application of unprecedented large-scale quarantine measures that essentially confine residents to their homes and is likely to have a negative psychosocial effect on residents [4]. Furthermore, a unique “infodemic”—an overabundance of (mis)information on social media [5] and elsewhere—poses a major risk to public mental health during this health crisis. It has become evident that psychiatric disorders, such as depression, anxiety, and posttraumatic stress disorder, developed in high-risk persons, especially survivors and frontline healthcare workers providing guiding principles for emergency psychological crisis interventions to reduce the psychosocial effects of the COVID-19 outbreak [5].

COVID-19 infection results in the secretion of cytokines and the activation of the kynurenine pathway of tryptophan metabolism. These chemicals enhance the disturbance in the limbic circuits which can be linked with psychiatric disorders such as psychoses, bipolar disorder (BD), depression, and suicide [6]. The actual effect of the virus on the brain and possible psychiatric manifestations is still an area of study and further investigation. There are also several case reports showing manic like symptoms after COVID-19 infection [7]. The COVID-19 pandemic has had a profound effect on all aspects of society, including mental health and physical health [8]. It is a fact that the direct and indirect psychological and social effects of the coronavirus disease 2019 (COVID-19) pandemic are pervasive and could affect mental health now and in the future [9].

We describe the case of a 55-year-old patient who presented with behavioral and mood symptoms after a COVID-19 infection and discuss the issues raised regarding the psychiatric manifestation and the treatment implications for his illness. This case might explain the link between COVID-19 infection and manic-like symptoms.

## 2. Main Body of Text

### 2.1. Case Presentation

AT is a 55-year-old male patient who came from “Serebo.” He is a married father who has four children. He was a high school Amharic teacher until his illness. He came to our setup with his son in law, and history was taken from the patient himself with no language barrier. The information obtained is reliable. He came to our hospital with a referral. He had no previous history of mental illness, no family history of mental or suicidal history, and no substance use history; this was his first visit and first admission to the Jimma University Medical Center, Department of Psychiatry.

He was a known hypertensive patient for the last 1 year. He was on medication (hydrochlorothiazide 25 mg) and follow-up at our medical center, and his blood pressure was controlled. The patient was in a relatively healthy and functional state until 2 weeks back before he experienced cough, high-grade fever, and shortness of breath. For these complaints, he visited a local clinic where he was diagnosed with typhoid fever. However, his symptoms persisted even after typhoid treatment (ciprofloxacin 500 mg bid for 7 days), and he was referred to Jimma University Medical Center for COVID-19 testing and further evaluation. Then, he was found to be positive for COVID-19 and was admitted to the COVID-19 treatment center at the Shenen Gibe Primary Hospital, where he was treated with intranasal O_2_, dexamethasone, and azithromycin and multivitamin supplement. At this time, he was having behavioral disturbance before the first dose of dexamethasone and azithromycin.

### 2.2. Current Presentation

Starting from a day before his diagnosis of COVID-19 and admission, he started to display bizarre behavioral change. As the patient reported, he was denying the existence of the virus and his test result. Saying, “It is not a real virus, and even if it is real, it cannot affect me in any way.” He noted that the situation by then was stressful; he mainly mentioned many problems in his treatment process beginning from his harsh ambulance trip to the corona center.

He claims that COVID-19 cannot harm him in any way since he is a special spiritual person, even after his symptoms like his shortness of breath worsened to the level he required oxygen support to breath he was having an euphoric mood and extensive confidence about himself.

He also criticized the understandard treatment he got in the center saying “It was very irritating, it was not the care I deserved and they were not qualified for me.” And he also added he is capable of making the center a better place, single handedly. He even mentioned staying late in the night and cleaning the toilet of the center by himself multiple times. In addition, he also starts a fight with one of the nurses in the facility, blaming him for not working fast enough. He threatened and chased the nurse with a broom stick before the hospital guards stopped him. He accepted the fact that he was becoming irritated with no provocation, very talkative to the extent that he felt pain to the corner of his lips. He was talking a lot with strangers and called a number of people on the phone, finishing all his money for phone call credits.

He also noted he had decreased need for sleep and was very energetic to fix things around him even at the time he was on oxygen. The family also reported that the patient had become over talkative, and they were unable to catch-up with him. When asked about the content of his speech they say he talks about a number of issues including abilities and criticism, they heard him repeatedly saying, “I am a very special person, and corona can't affect me.” They also noted that he was very irritable literally on anything. In addition, he was also extravagant on his phone call spending. They said the elicited symptoms of the illness shortly grew more severe, significantly affecting his functionality, interpersonal relationships, and his engagement with his social circle; and after his second test of COVID-19 became negative, he was referred to our psychiatry set-up.

### 2.3. Laboratory Investigation

He was diagnosed with COVID-19 infection using PCR. Routine laboratory tests including CBC, WBC 20,600 *μ*/L, and ESR 42 mm/hr were abnormal. The remaining routine laboratory tests were in the normal range including Hct (47.5%), PLT (336,000), TSH (3.5 uU/mL), CRP (6.6 mg/L), RBS (100 mg/dL), Serum Electrolyte (Na+141 mmol/L, K+4.14 mmol/L, Cl 106.8 mmol/L), LFT (ASTL 59.6 U/L, ALT 57.9 U/L), and RFT (Creatine 0.98). However, we did not perform a brain biopsy to establish the cause-effect relationship of COVID-19 infection of the brain.

### 2.4. Neuropsychological Evaluation

Neuropsychological evaluation at admission to the psychiatric ward showed psychomotor agitation. The speech was loud, rapid, and pressured. There were grandiose delusions and flight of ideas. He had no suicidal/homicidal ideation. He is alert and oriented to place, person, and time. The fund of knowledge and abstract thinking was intact. He had good concentration and memory.

### 2.5. Course of the Illness and Management

The patient presented with behavioral disturbance after a diagnosis of COVID-19. He exhibited symptoms including irritability, verbal and physical aggressiveness, increased goal-directed activity, elated and expansive mood, increased energy, grandiosity and inflated self-esteem, and decreased need for sleep.

With the diagnosis of bipolar I disorder, current manic episode, severe, with psychotic feature, and first manic episode due to COVID-19 infection, he was admitted to the psychiatry emergency ward and started on a loading dose of 1000 mg sodium valproate and put on a standing dose of 500 mg po sodium valporate bid and haloperidol (5 mg/day) with 10 mg po diazepam.

By day 6, he developed extrapyramidal side effects. For it, we tapered haloperidol to 2.5 mg. With this management, he becomes symptom free by day 8, and on the same day, COVID-19 test was done, and it becomes negative. By day 13, he was transferred to the cold ward with significant improvement taking sodium valproate 500 mg po bid and haloperidol 2.5 mg po, and later, haloperidol stopped. By day 21, he was discharged from the hospital with full improvement and received sodium valproate 500 mg po bid as a monotherapy. In addition, psycho-education was given to patients and family members on the nature of illness and importance of drug adherence. See Table 1 for the details of patient management in the emergency ward.

## 3. Discussion

In this case, we present a 55-year-old patient with no previous mental illness background who presented with full-blown manic symptoms following a severe course of COVID-19 infections that required admission. According to the presentation of the patient and our meticulous observation, we came to the term that the new onset of manic symptoms that appeared in our patient can be attributed to COVID-19. Not only does the temporal relationship, absence of family history of mental illness, and unusual age of onset speak for the bipolar disorder secondary to medical condition (COVID-19 infection), but the nature of the virus in directly affecting the brain might be one explanation for the direct effect of the virus in the CNS. One explanation could be the direct effect of viruses in the CNS. Evidence shows that a case of meningitis/encephalitis associated with SARS-CoV-2 was reported, and specific SARS-CoV-2 RNA was detected in CSF in a 24-year-old patient with COVID-19 [10]. Several lines of evidence have highlighted the neuroinvasive potential of SARS-CoV-2-induced CNS symptoms.

Another interpretation of the link between COVID-19 infection and manic-like symptoms in our patient could be the effect of inflammation. Previous findings have demonstrated that infection-associated immune activation and subsequent release of inflammatory factors is one of the potential pathogenic mechanisms of bipolar disorder (BD). Several lines of evidence suggest increased plasma levels of IL-6, IL-10, and CRP [7] in the acute phase of the illness which further suggests that COVID-19 can initiate a cascade of inflammation that might contribute to manic-like symptoms.

Several studies have recommended that physicians in COVID-19 pandemic hotspots with abnormal neurologic damage and mental abnormalities perform antibody tests for CSF, and RNA tests are necessary for patients with mental abnormalities following COVID-19. Long-term follow-up is recommended for these patients even if the RNA test for the virus shows negative results [11].

It is a fact that the mental health consequences of COVID-19 are partially determined by an interaction between precision brains profiles and the universal threat to human connection posed by the virus. It is useful to conceptualize the mental health consequences of the pandemic as it captures the depth of COVID-19's threat to the human experience and generates hypotheses on the mental health implications of COVID-19 from a nuanced, precision psychiatry perspective [12].

One of the top differential diagnoses in our case was delirium secondary to medical condition; however, despite the acute onset, the patient was not confused or inattentive. In addition, he was oriented to time, place, and person. In addition, these all spoke against delirium. The other possible differential diagnoses bipolar disorder secondary to medication (corticosteroids). The fact that the symptoms of mania started before the diagnosis of COVID-19. The onset of manic symptoms precedes the start of dexamethasone. Studies suggest that manic episodes are the most common manifestations after of corticosteroid intake [13]. However, psychotic features or symptoms are prominent among patients with mania [13]. In contrast to this, the prominent symptoms on our patient is manic symptoms which goes against the existing evidences. The evidences suggest that there is a dose-dependent relationship between steroids, neuropsychiatric symptoms with higher doses than to low dose [14]. In our case, the use of low does and short-term (1 week) use can go against corticosteroid-induced mania.

The possibility of a primary bipolar affective disorder cannot be ruled out in our patient. He presented with classical features of mania after he was declared to have COVID-19 infection and was in clear sensorium at that time. Overall, the patient expressed his satisfaction with the treatment he received at our psychiatric setup. He is now relatively healthy and functional and attended his regular follow-up in our outpatient setting. In our assessment, this case report was able to establish the temporal relationship between COVID-19 infection and bipolar illness. However, this study lacks an established cause-effect relationship. Therefore, we would like to conclude that this patient is one of the samples that might suggest a direct effect of COVID-19 in causing bipolar disorder and encourage further studies on this topic.

### 3.1. Limitation

The strength of this case study is thorough, and detailed history was taken to ascertain the temporality of mania and COVID-19. The present study has the listed limitations. First, this is a single-case report, and the assumptions raised must be confirmed with future studies. In that case, the limitation of the present study was that the RT-PCR test for COVID-19 in CSF was not performed. In addition, neuroimaging was not performed to see any positive findings because of unaffordability.

## 4. Conclusion

The actual effect of COVID-19 on the brain and possible psychiatric manifestations is still an area of study and further investigation. There are also several case reports showing manic-like symptoms after COVID-19 infection. This case highlights the importance of paying attention to psychiatric symptoms in patients with COVID-19 and the early intervention and involvement of psychiatrists especially in critically ill patients. We also recommend performing antibody tests for CSF and RNA tests for patients with mental abnormalities following COVID-19. In the present scenario, we urge physicians to pay attention to those cases and open his/her eyes for such a possible new diagnosis. Further studies can be performed to identify the relationship between COVID-19 and bipolar disorders.

## Figures and Tables

**Table 1:** The course of illness and management of the patient in the emergency ward.

Day	Type and dose of medication	Main change in symptoms	Main change in MSE	Laboratory finding	Remark
1	Sodium valproate loading dose given, followed by sodium valproate 500 BID, haloperidol 5 mg/day, diazepam 5 mg nocte	Aggressive behavior decreased	Agitation decreased	CBC, LFT, RFT, TFT, RBS & ESR done	Tried to abscond and PRN given
2	Sodium valproate 500 BID, haloperidol 5 mg/day, diazepam 5 mg noct.	Sleep pattern started improving	No longer agitated		
3	Sodium valproate 500 BID, haloperidol 5 mg/day	Energy decreased	No longer agitated		Diazepam stopped
4	Sodium valproate 500 BID, haloperidol mg/day	Energy decreased	Flight of ideas decreased		
5	Sodium valproate 500 BID, haloperidol 5 mg/day	Elated mood lowered	Flight of ideas decrease		
6	Sodium valproate 500 BID, haloperidol 5 mg/day	Elated mood lowered	Flight of ideas decrease		Hand tremor
7	Sodium valproate 500 BID, haloperidol 2.5 mg/day	Elated mood lowered	Grandiose delusion decreased	CBC, ESR normal	Haloperidol decreased by 2.5 mg
8	Sodium valproate 500 BID, haloperidol 2.5 mg/day	Manic symptoms subsided	No delusion		COVID-19 test became negative
9	Sodium valproate 500 BID, haloperidol 2.5 mg/day	Manic symptoms subsided	No delusion		
10	Sodium valproate 500 BID, haloperidol 2.5 mg/day	Manic symptoms subsided	No delusion		
11	Sodium valproate 500 BID, haloperidol 2.5 mg/day	Manic symptoms subsided	No delusion		Hand tremor subsided
12	Sodium valproate 500 BID, haloperidol 2.5 mg/day	Manic symptoms subsided	No delusion		
13	Sodium valproate 500 BID, haloperidol 2.5 mg/day	Manic symptoms subsided	No delusion		Patient transferred to cold ward

## Abbreviations

COVID-19: Coronavirus disease 2019
CRP: C-reactive protein
LFT: Liver function test
MSE: Mental status examination
RFT: Renal function test
SARS-CoV-2: Severe acute respiratory syndrome coronavirus 2
MSE: Mental status examination.
